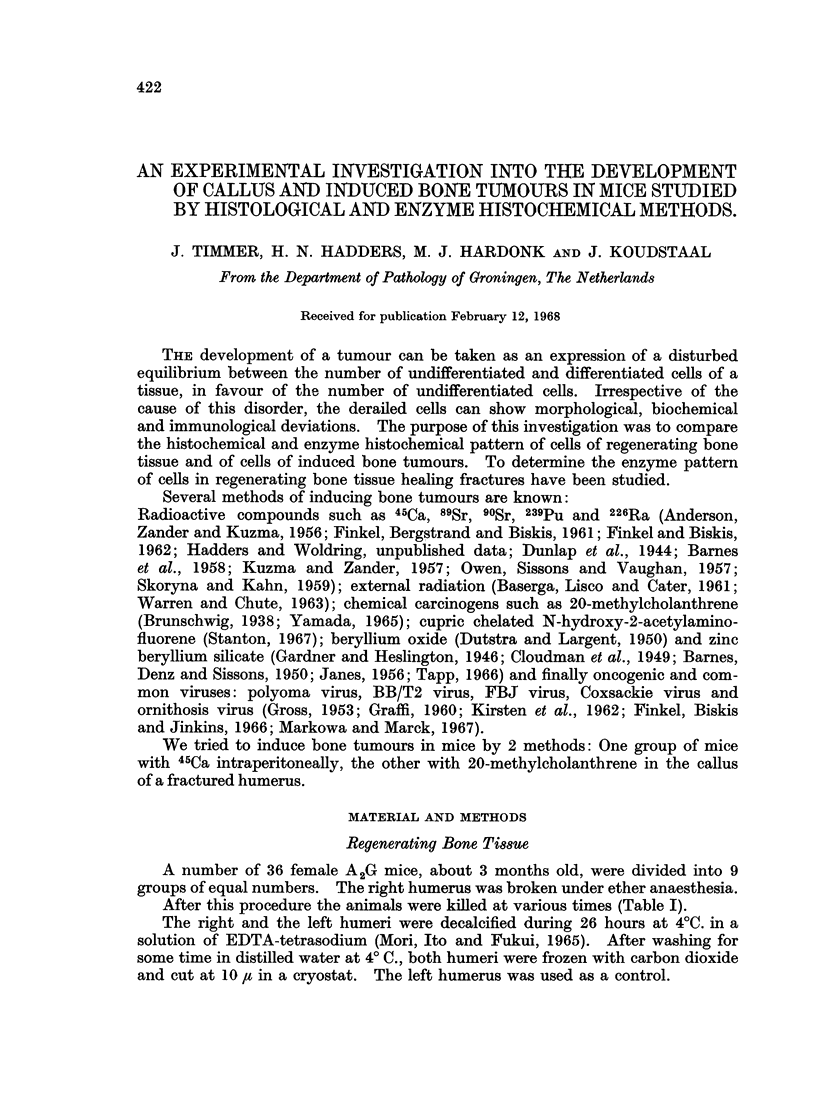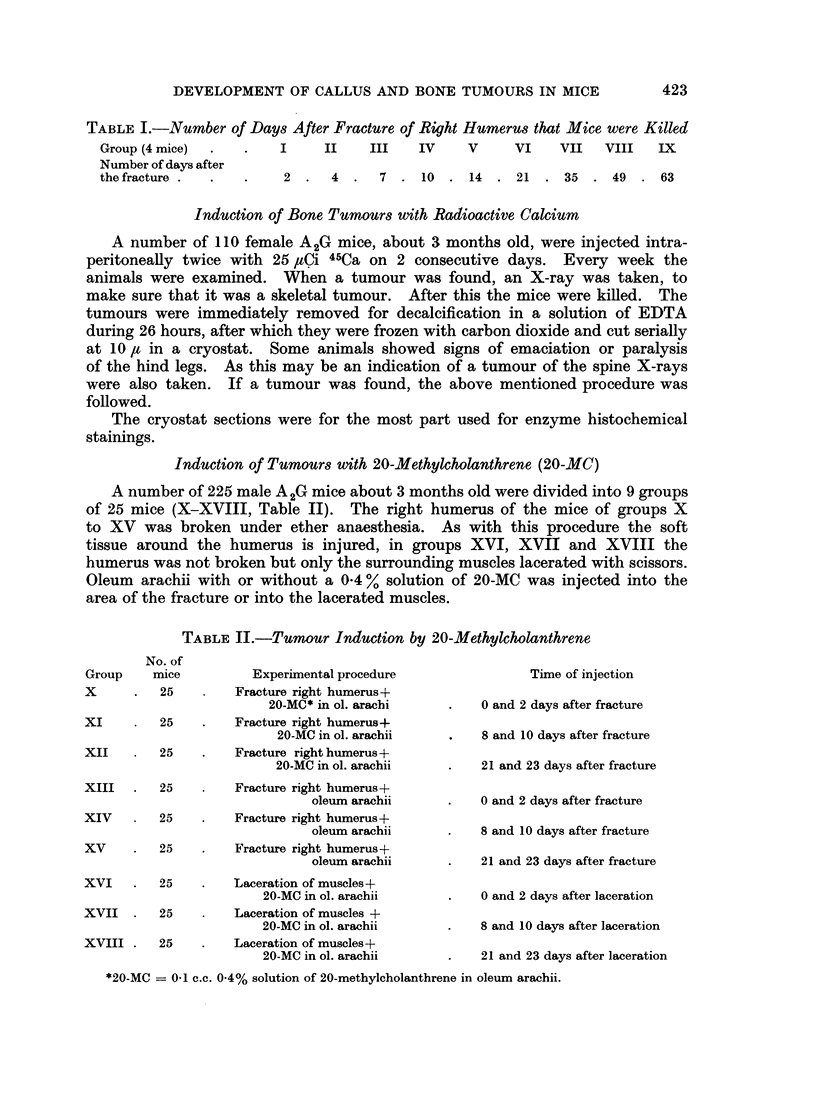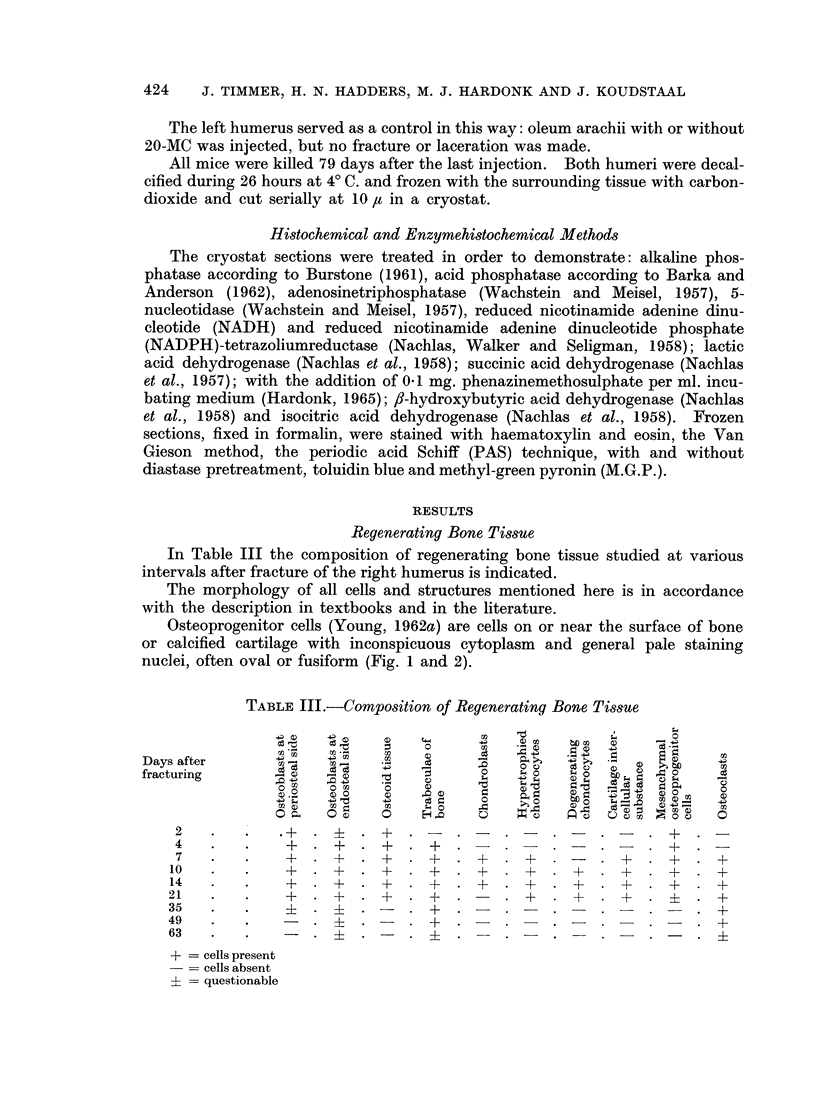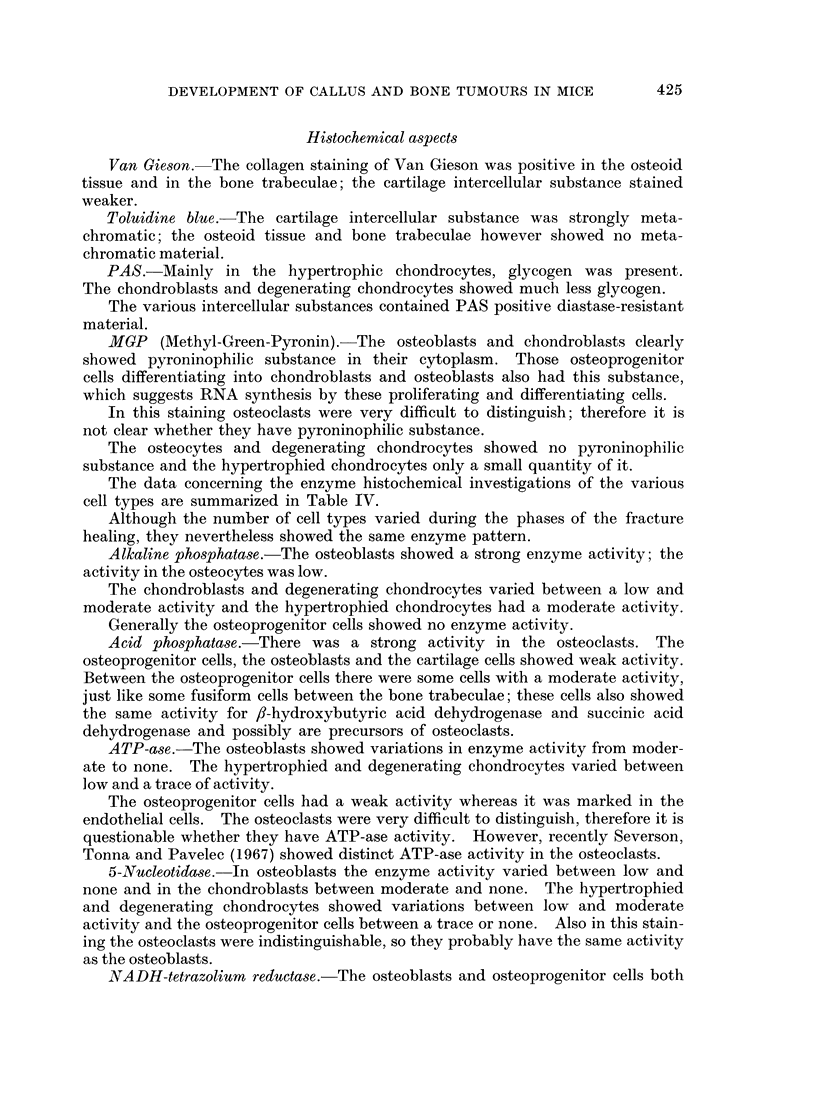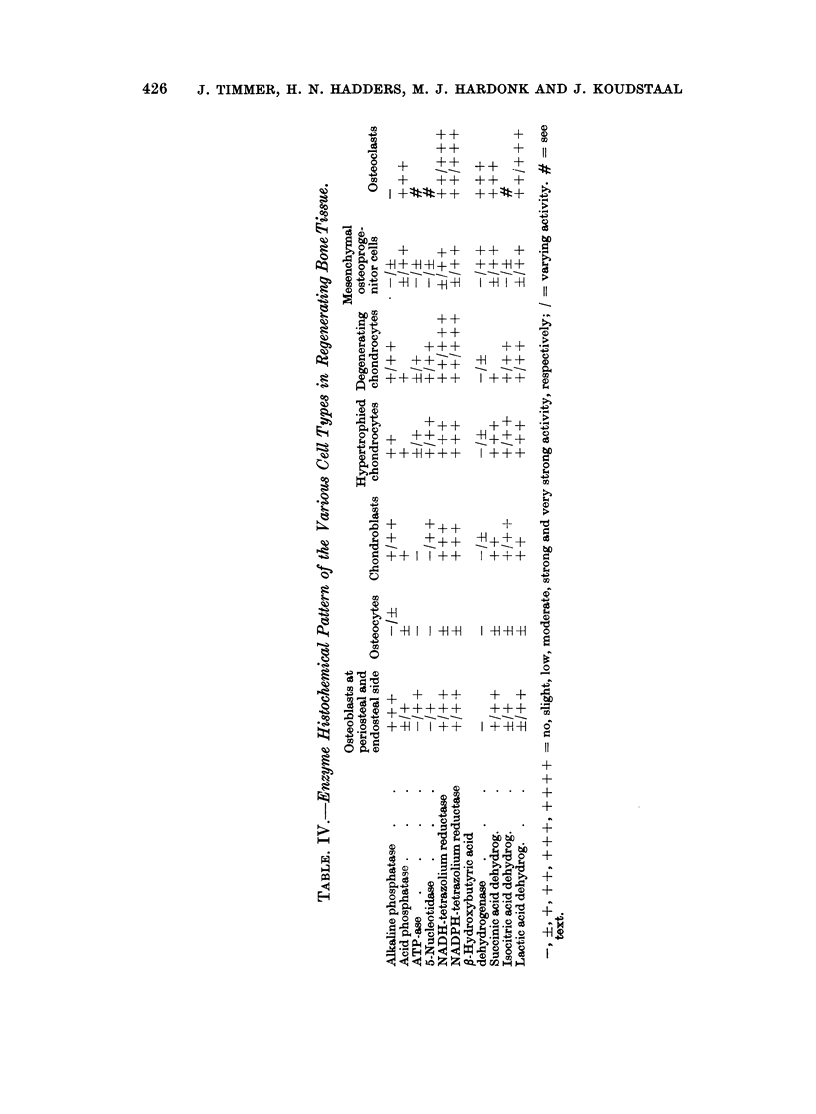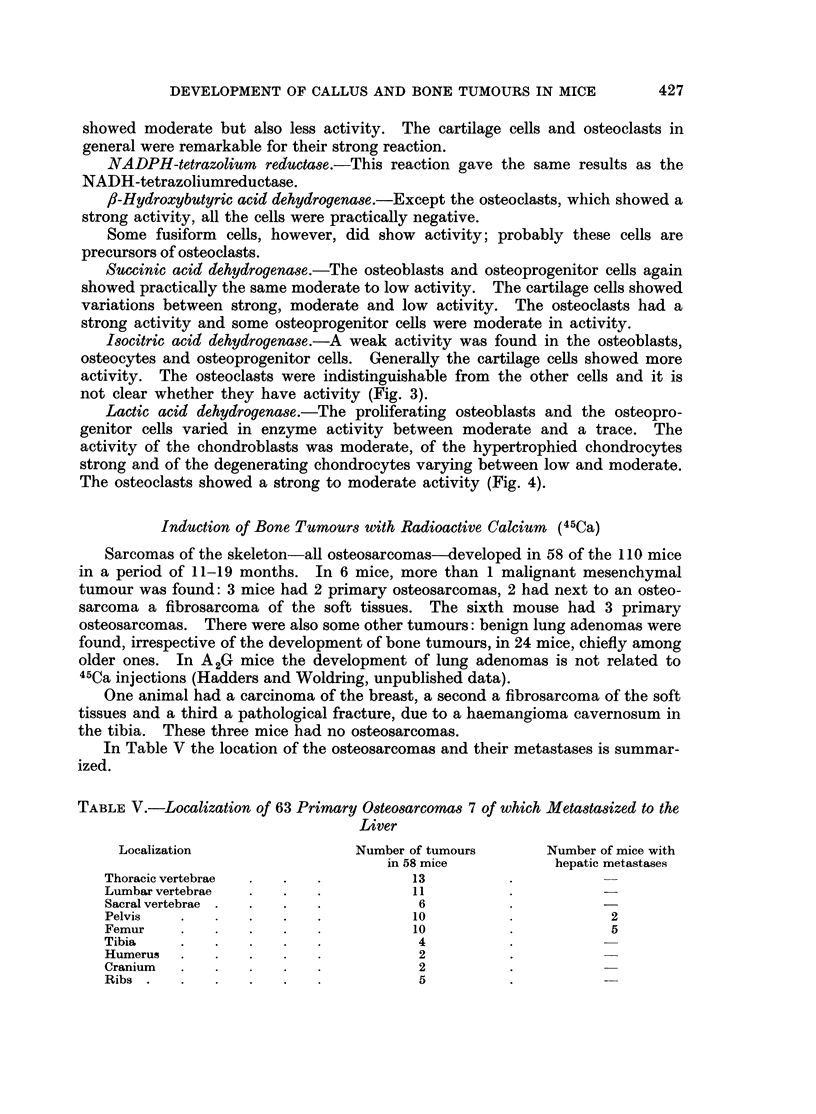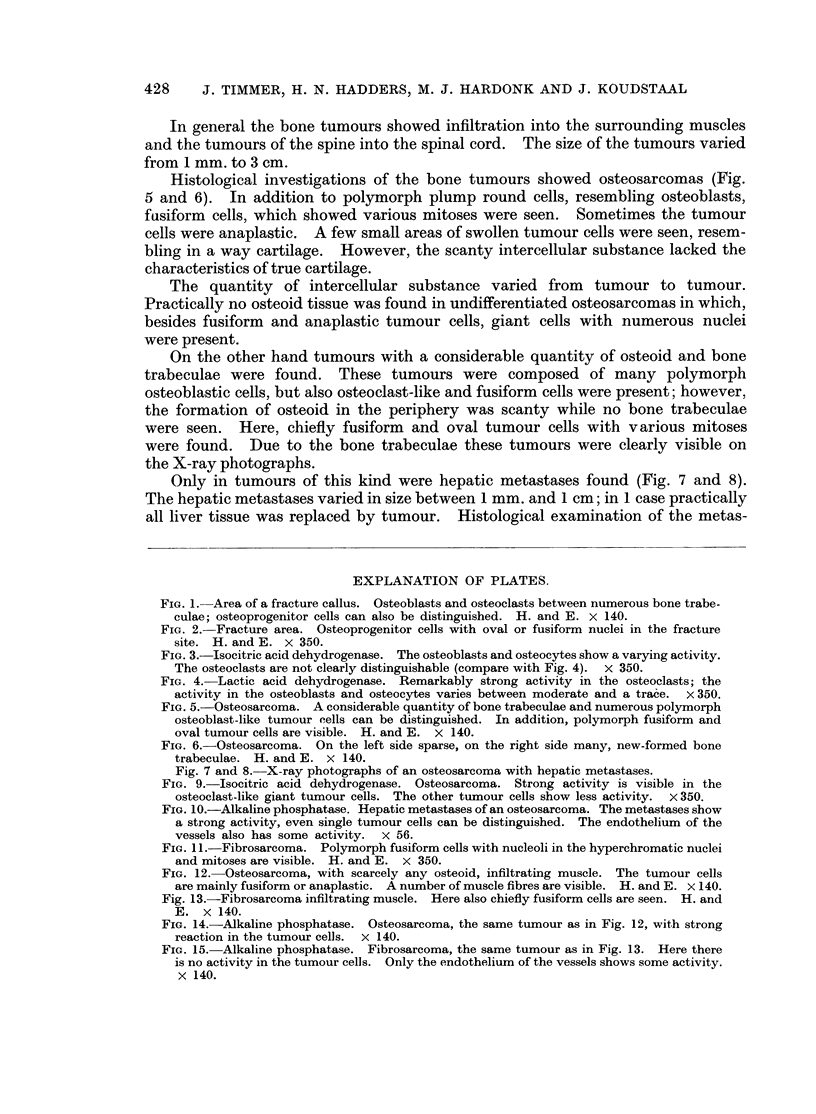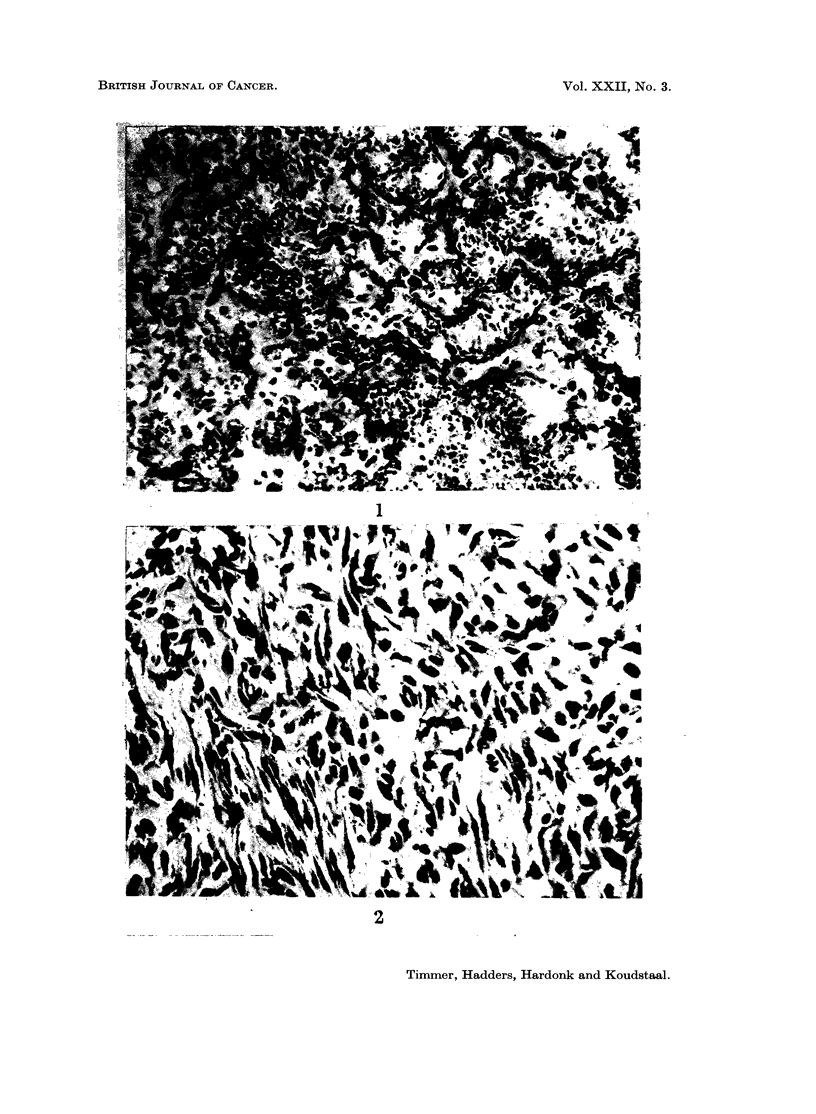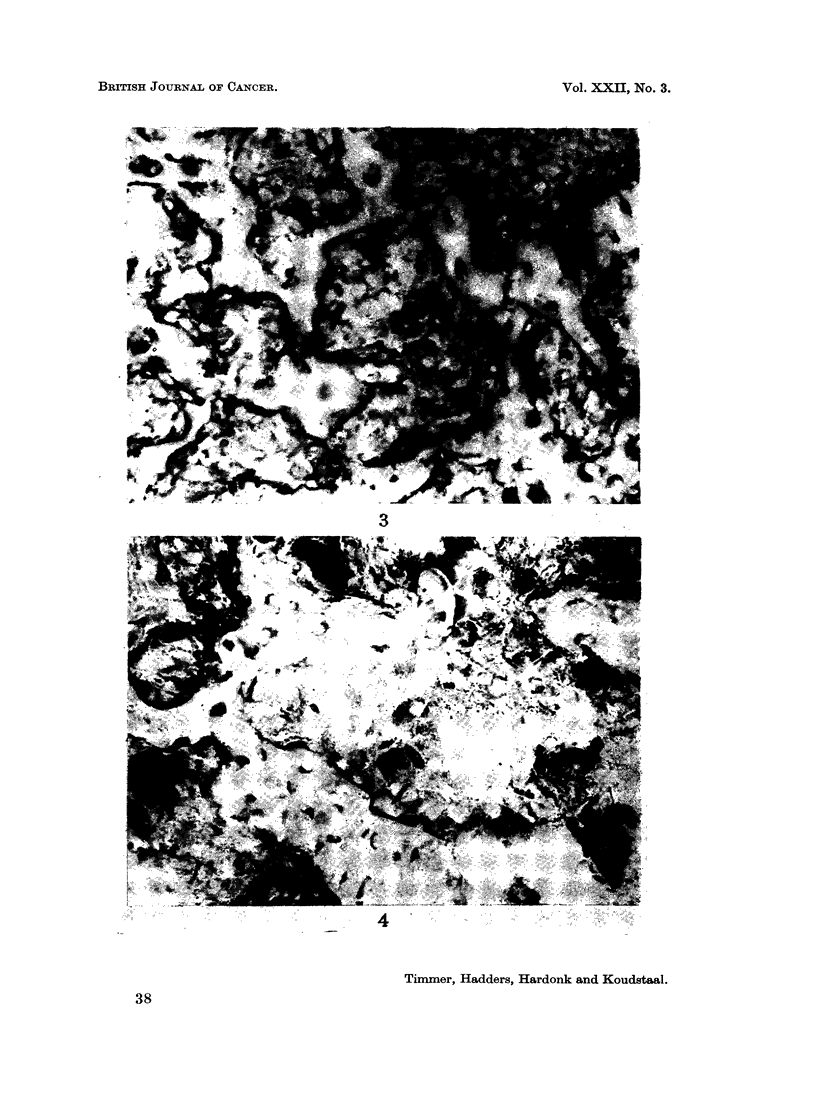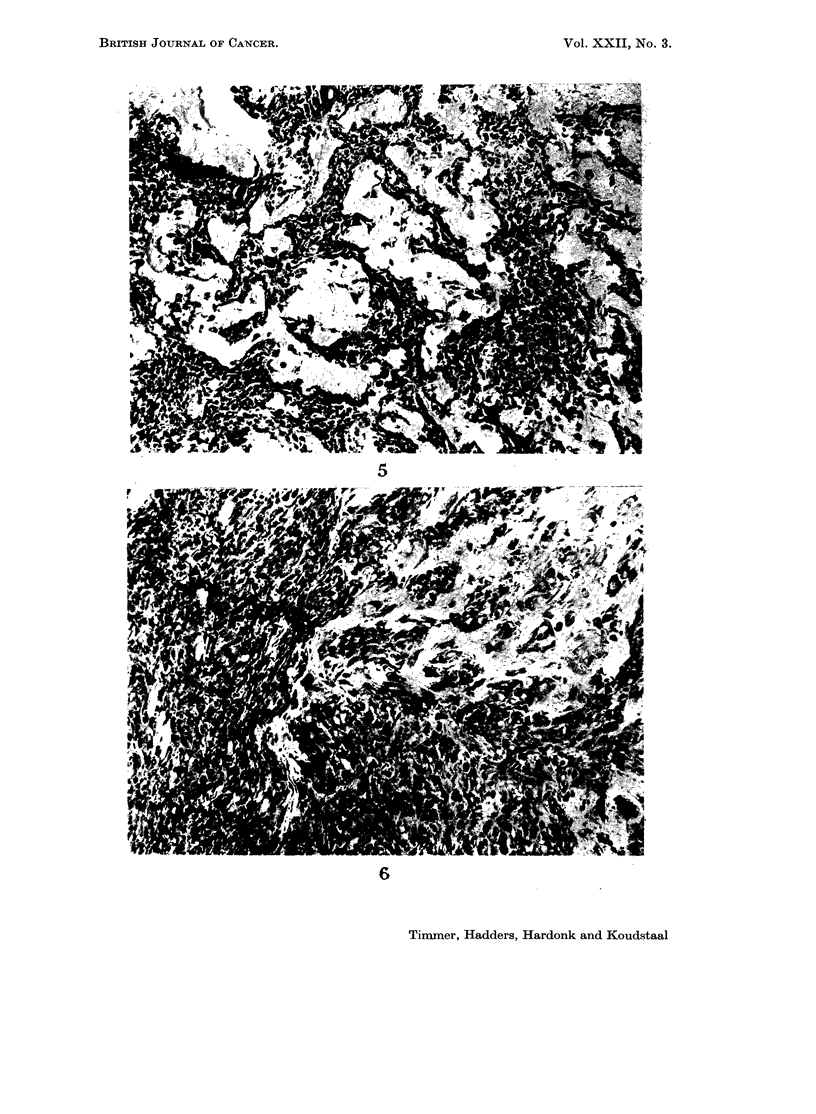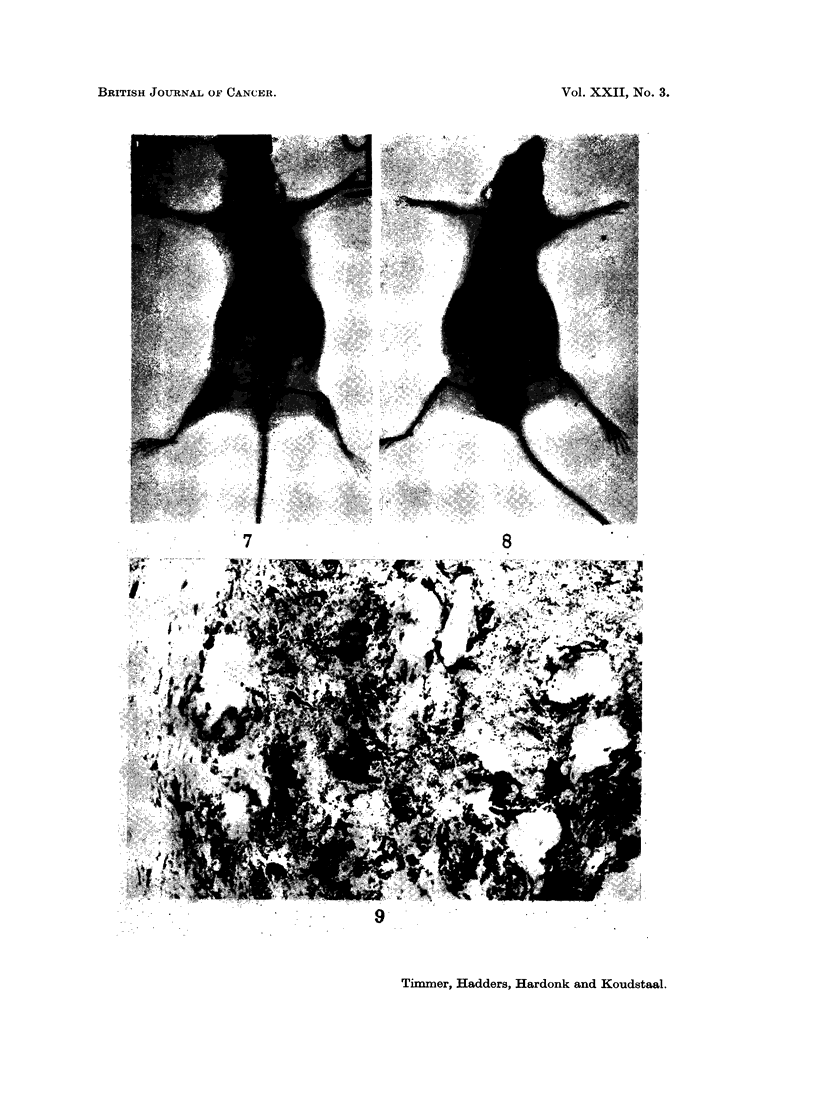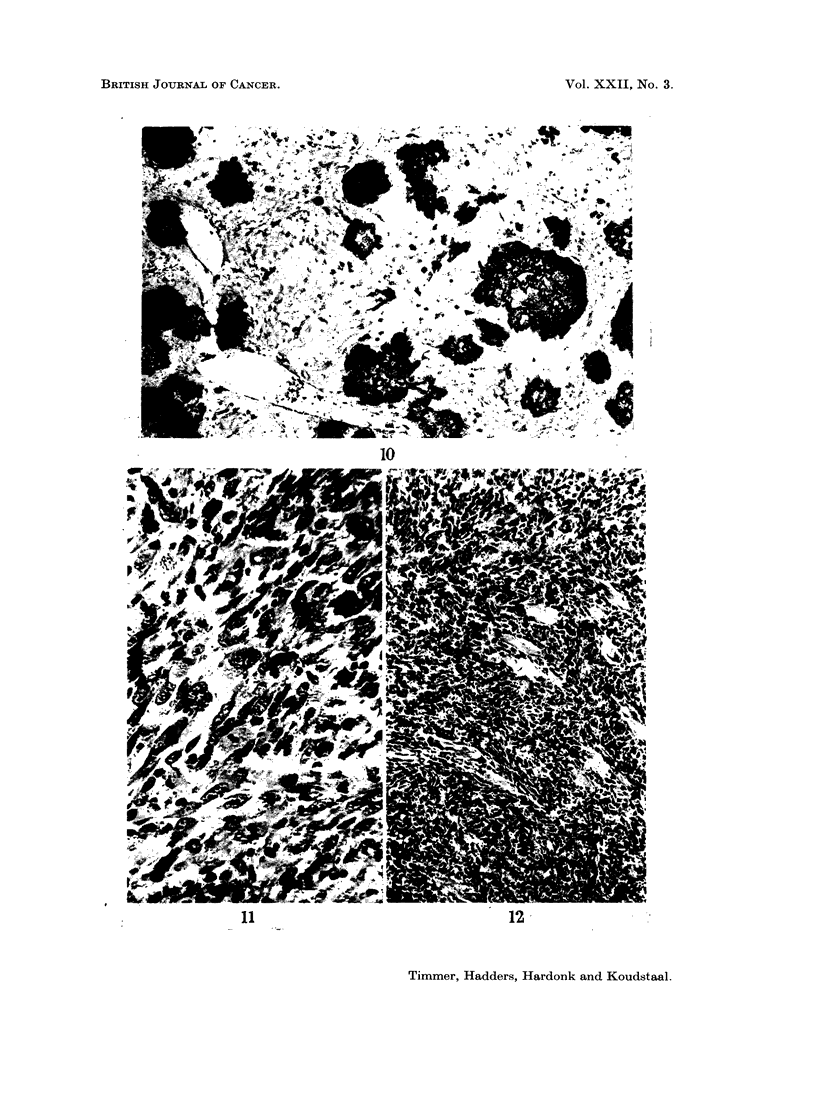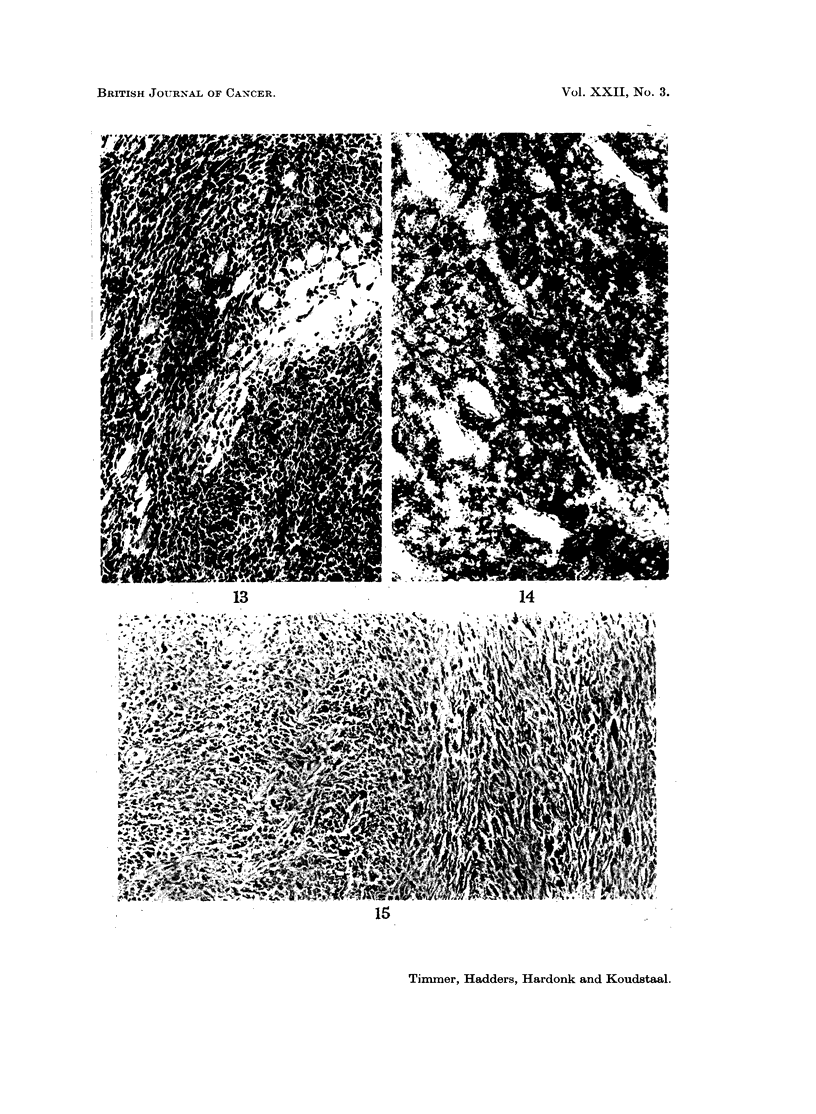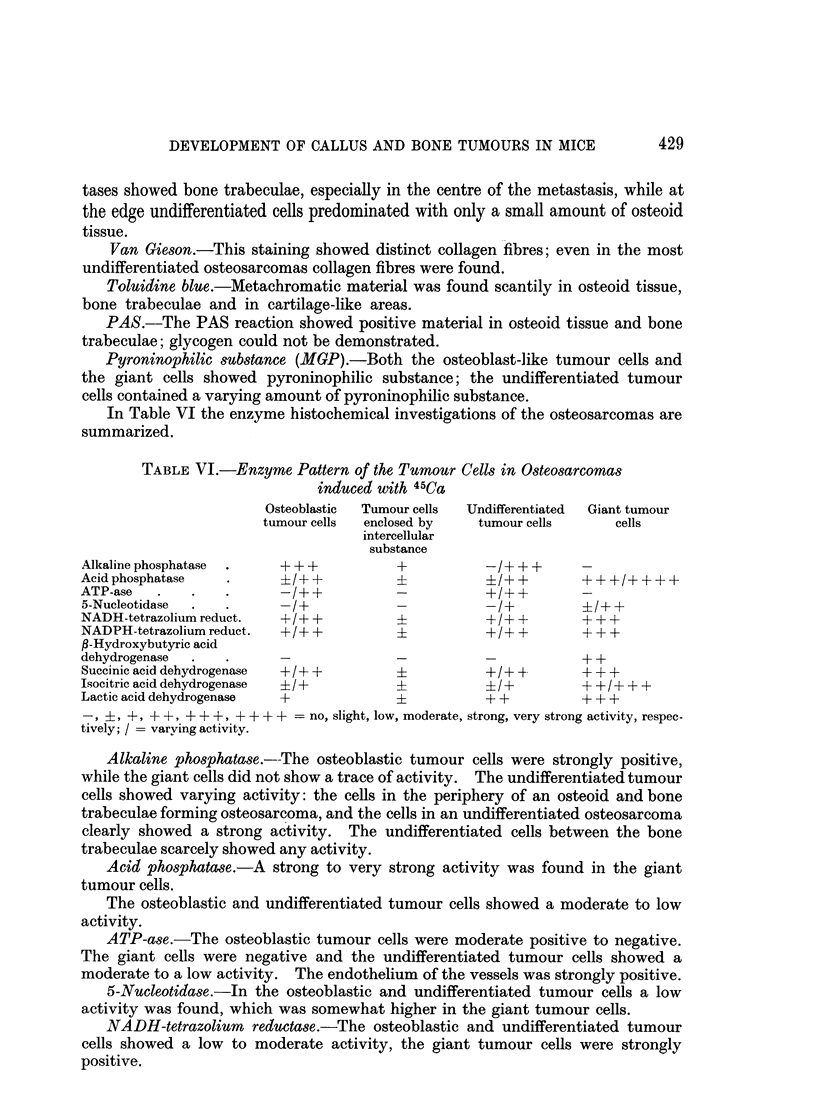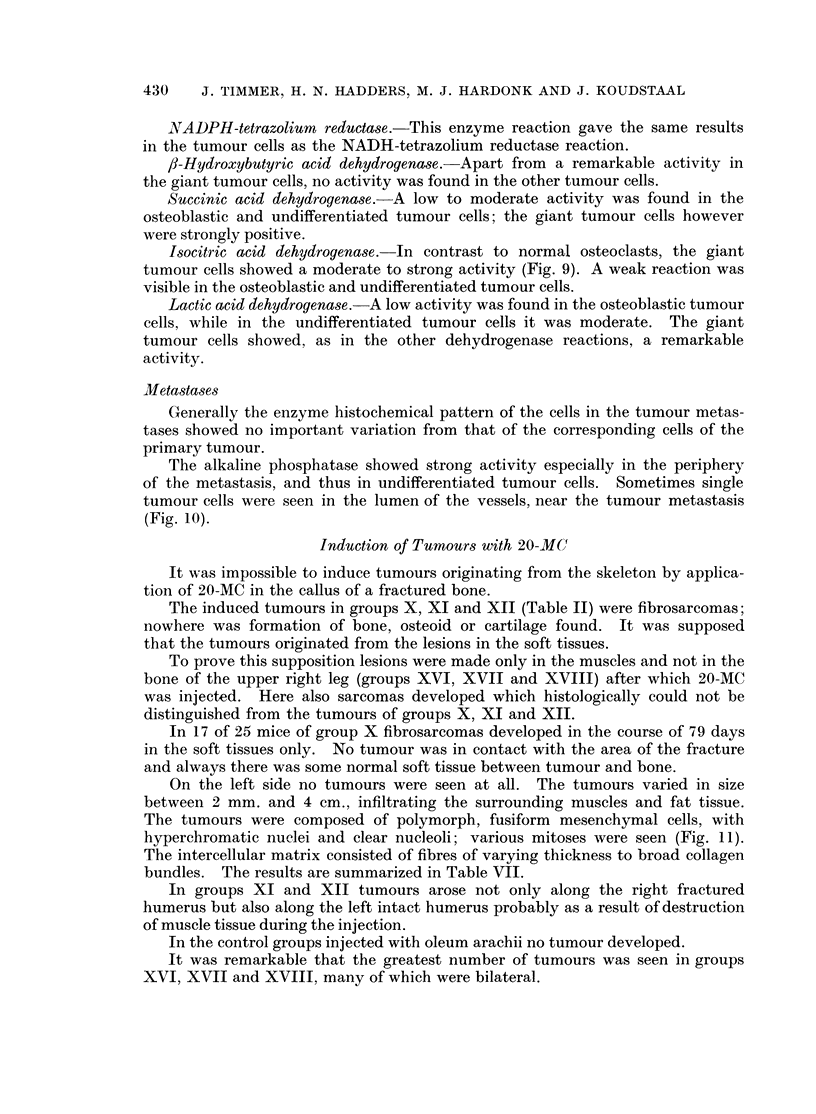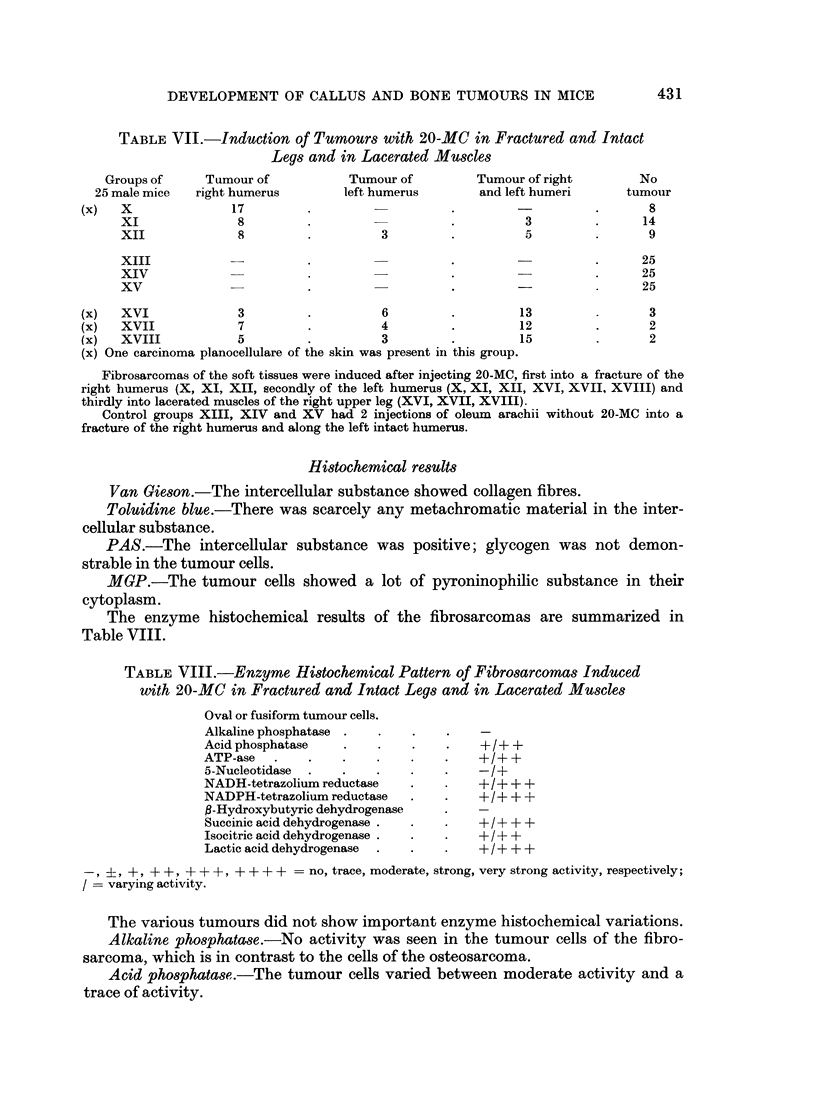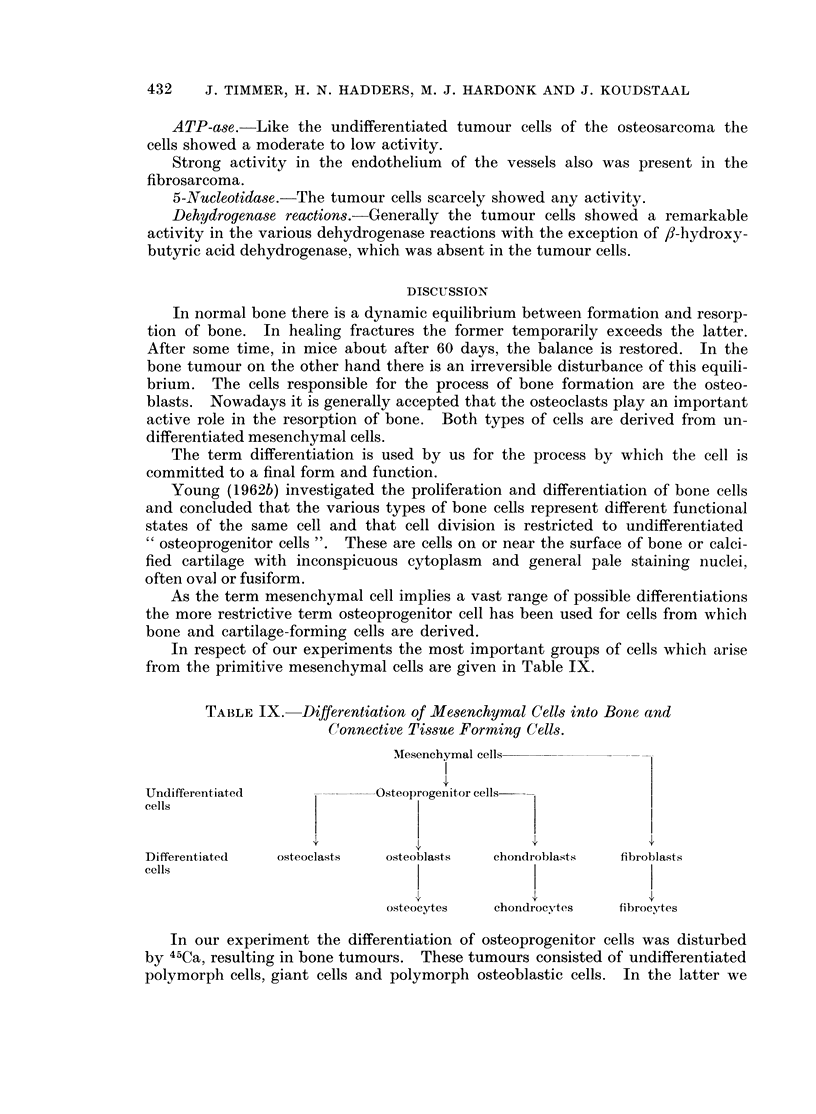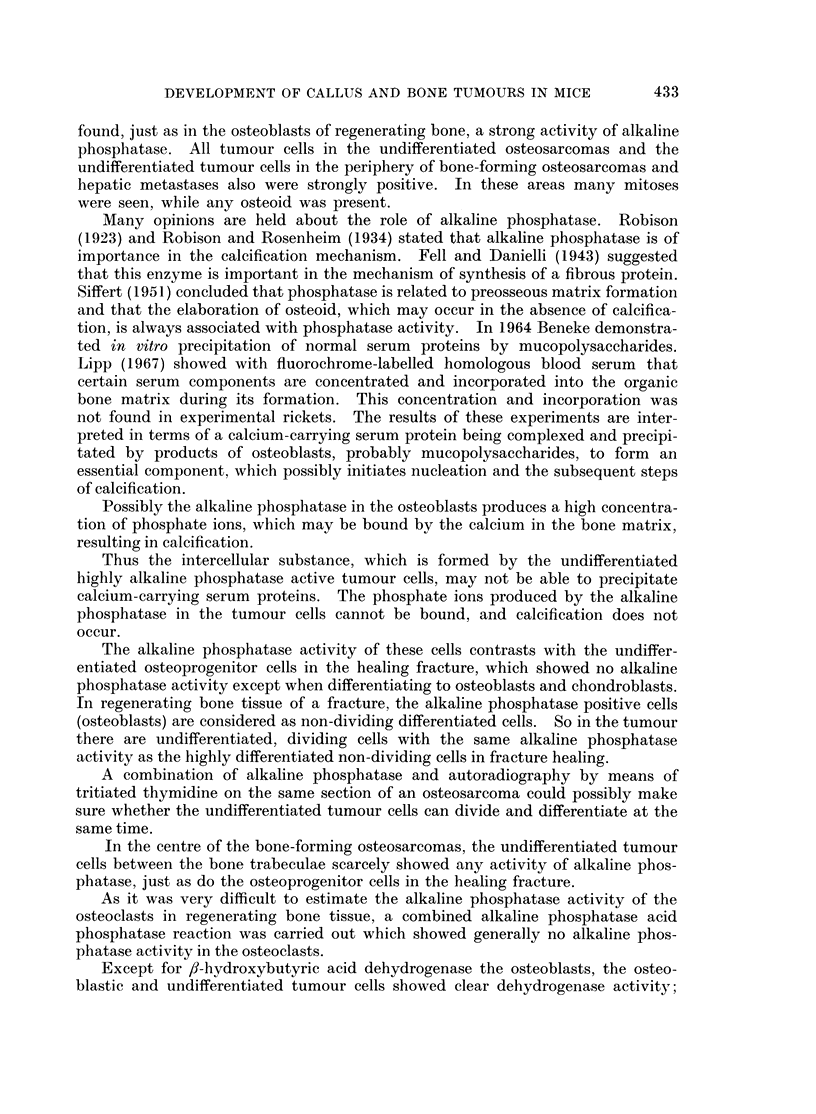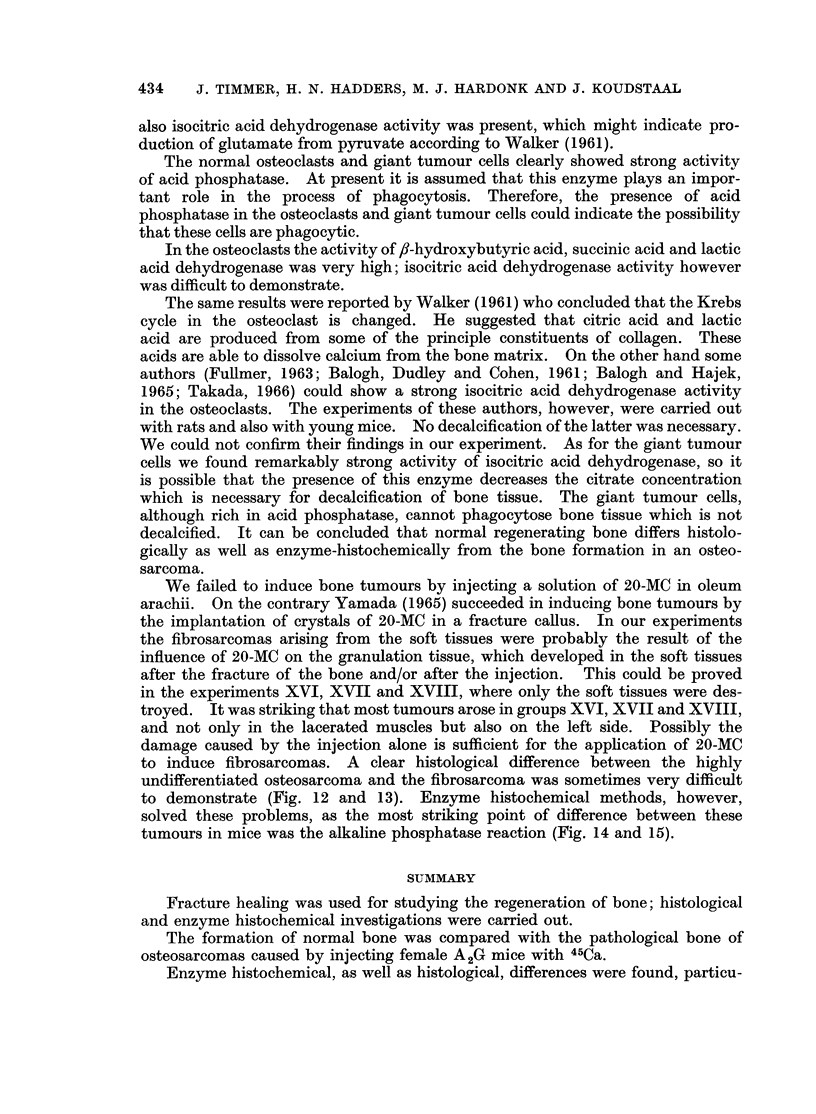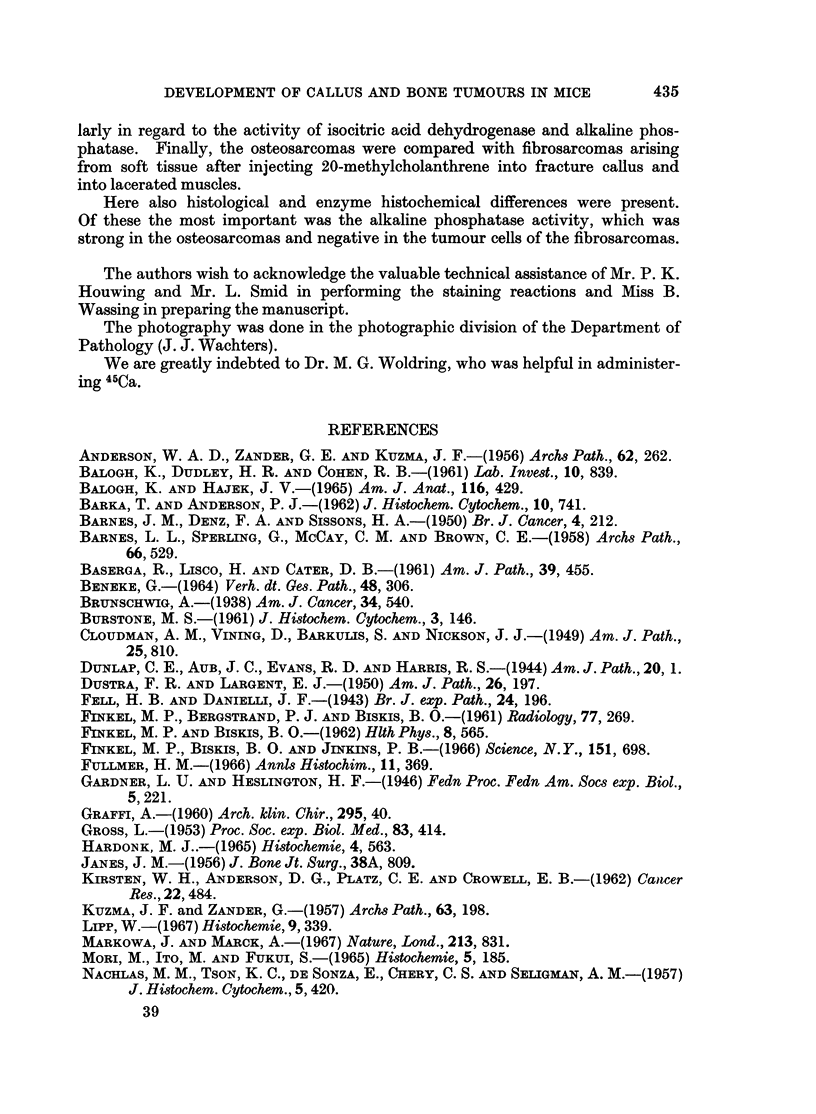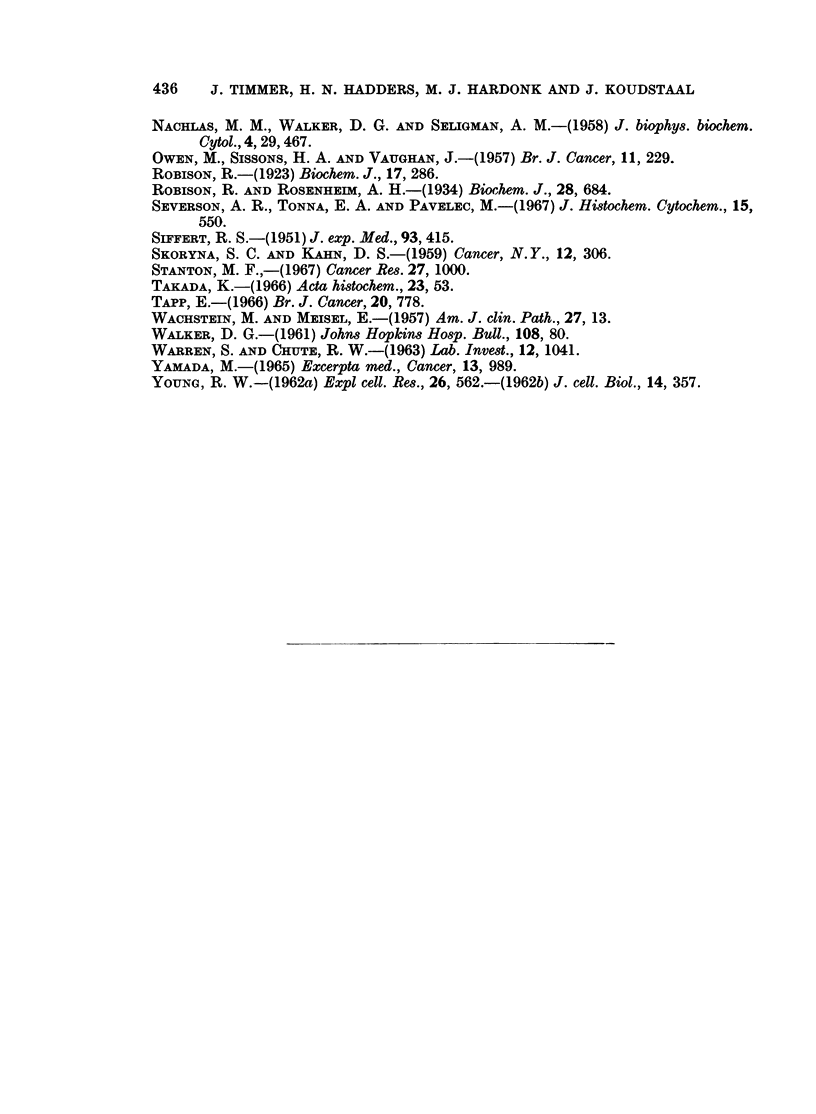# An experimental investigation into the development of callus and induced bone tumours in mice studies by histological and enzyme histochemical methods.

**DOI:** 10.1038/bjc.1968.50

**Published:** 1968-09

**Authors:** J. Timmer, H. N. Hadders, M. J. Hardonk, J. Koudstaal

## Abstract

**Images:**


### AN EXPERIMENTAL INVESTIGATION INTO THE DEVELOPMENT OF CALLUS AND INDUCED BONE TUMOURS IN MICE STUDIED BY HISTOLOGICAL AND ENZYME HISTOCHEMICAL METHODS.

J. TIMMER, H. N. HADDERS, M. J. HARDONK AND J. KOUDSTAAL

*From the Department of Pathology of Groningen, The Netherlands*

Received for publication February 12, 1968

THE development of a tumour can be taken as an expression of a disturbed equilibrium between the number of undifferentiated and differentiated cells of a tissue, in favour of the number of undifferentiated cells. Irrespective of the cause of this disorder, the derailed cells can show morphological, biochemical and immunological deviations. The purpose of this investigation was to compare the histochemical and enzyme histochemical pattern of cells of regenerating bone tissue and of cells of induced bone tumours. To determine the enzyme pattern of cells in regenerating bone tissue healing fractures have been studied.

Several methods of inducing bone tumours are known:
Radioactive compounds such as $^{45}$Ca, $^{89}$Sr, $^{90}$Sr, $^{239}$Pu and $^{226}$Ra (Anderson, Zander and Kuzma, 1956; Finkel, Bergstrand and Biskis, 1961; Finkel and Biskis, 1962; Hadders and Woldring, unpublished data; Dunlap *et al.*, 1944; Barnes *et al.*, 1958; Kuzma and Zander, 1957; Owen, Sissons and Vaughan, 1957; Skoryna and Kahn, 1959); external radiation (Baserga, Lisco and Cater, 1961; Warren and Chute, 1963); chemical carcinogens such as 20-methylcholanthrene (Brunschwig, 1938; Yamada, 1965); cupric chelated N-hydroxy-2-acetylamino-fluorene (Stanton, 1967); beryllium oxide (Dutstra and Largent, 1950) and zinc beryllium silicate (Gardner and Heslington, 1946; Cloudman *et al.*, 1949; Barnes, Denz and Sissons, 1950; Janes, 1956; Tapp, 1966) and finally oncogenic and common viruses: polyoma virus, BB/T2 virus, FBJ virus, Coxsackie virus and ornithosis virus (Gross, 1953; Graffi, 1960; Kirsten *et al.*, 1962; Finkel, Biskis and Jinkins, 1966; Markowa and Marck, 1967).

We tried to induce bone tumours in mice by 2 methods: One group of mice with $^{45}$Ca intraperitoneally, the other with 20-methylcholanthrene in the callus of a fractured humerus.

### MATERIAL AND METHODS

### *Regenerating Bone Tissue*

A number of 36 female $A_2G$ mice, about 3 months old, were divided into 9 groups of equal numbers. The right humerus was broken under ether anaesthesia.

After this procedure the animals were killed at various times (Table I).

The right and the left humeri were decalcified during 26 hours at 4°C. in a solution of EDTA-tetrasodium (Mori, Ito and Fukui, 1965). After washing for some time in distilled water at 4° C., both humeri were frozen with carbon dioxide and cut at 10 $\mu$ in a cryostat. The left humerus was used as a control.

TABLE I.—*Number of Days After Fracture of Right Humerus that Mice were Killed*

| Group (4 mice) | I | II | III | IV | V | VI | VII | VIII | IX |
|---|---|---|---|---|---|---|---|---|---|
| Number of days after the fracture | 2 | 4 | 7 | 10 | 14 | 21 | 35 | 49 | 63 |

### *Induction of Bone Tumours with Radioactive Calcium*

A number of 110 female $A_2G$ mice, about 3 months old, were injected intraperitoneally twice with 25 $\mu$Ci $^{45}Ca$ on 2 consecutive days. Every week the animals were examined. When a tumour was found, an X-ray was taken, to make sure that it was a skeletal tumour. After this the mice were killed. The tumours were immediately removed for decalcification in a solution of EDTA during 26 hours, after which they were frozen with carbon dioxide and cut serially at 10 $\mu$ in a cryostat. Some animals showed signs of emaciation or paralysis of the hind legs. As this may be an indication of a tumour of the spine X-rays were also taken. If a tumour was found, the above mentioned procedure was followed.

The cryostat sections were for the most part used for enzyme histochemical stainings.

### *Induction of Tumours with 20-Methylcholanthrene (20-MC)*

A number of 225 male $A_2G$ mice about 3 months old were divided into 9 groups of 25 mice (X–XVIII, Table II). The right humerus of the mice of groups X to XV was broken under ether anaesthesia. As with this procedure the soft tissue around the humerus is injured, in groups XVI, XVII and XVIII the humerus was not broken but only the surrounding muscles lacerated with scissors. Oleum arachii with or without a 0·4% solution of 20-MC was injected into the area of the fracture or into the lacerated muscles.

TABLE II.—*Tumour Induction by 20-Methylcholanthrene*

| Group | No. of mice | Experimental procedure | Time of injection |
|---|---|---|---|
| X | 25 | Fracture right humerus + 20-MC* in ol. arachi | 0 and 2 days after fracture |
| XI | 25 | Fracture right humerus + 20-MC in ol. arachii | 8 and 10 days after fracture |
| XII | 25 | Fracture right humerus + 20-MC in ol. arachii | 21 and 23 days after fracture |
| XIII | 25 | Fracture right humerus + oleum arachii | 0 and 2 days after fracture |
| XIV | 25 | Fracture right humerus + oleum arachii | 8 and 10 days after fracture |
| XV | 25 | Fracture right humerus + oleum arachii | 21 and 23 days after fracture |
| XVI | 25 | Laceration of muscles + 20-MC in ol. arachii | 0 and 2 days after laceration |
| XVII | 25 | Laceration of muscles + 20-MC in ol. arachii | 8 and 10 days after laceration |
| XVIII | 25 | Laceration of muscles + 20-MC in ol. arachii | 21 and 23 days after laceration |

*20-MC = 0·1 c.c. 0·4% solution of 20-methylcholanthrene in oleum arachii.

The left humerus served as a control in this way: oleum arachii with or without 20-MC was injected, but no fracture or laceration was made.

All mice were killed 79 days after the last injection. Both humeri were decalcified during 26 hours at 4° C. and frozen with the surrounding tissue with carbon-dioxide and cut serially at 10 $\mu$ in a cryostat.

### *Histochemical and Enzymehistochemical Methods*

The cryostat sections were treated in order to demonstrate: alkaline phosphatase according to Burstone (1961), acid phosphatase according to Barka and Anderson (1962), adenosinetriphosphatase (Wachstein and Meisel, 1957), 5-nucleotidase (Wachstein and Meisel, 1957), reduced nicotinamide adenine dinucleotide (NADH) and reduced nicotinamide adenine dinucleotide phosphate (NADPH)-tetrazoliumreductase (Nachlas, Walker and Seligman, 1958); lactic acid dehydrogenase (Nachlas *et al.*, 1958); succinic acid dehydrogenase (Nachlas *et al.*, 1957); with the addition of 0·1 mg. phenazinemethosulphate per ml. incubating medium (Hardonk, 1965); $\beta$-hydroxybutyric acid dehydrogenase (Nachlas *et al.*, 1958) and isocitric acid dehydrogenase (Nachlas *et al.*, 1958). Frozen sections, fixed in formalin, were stained with haematoxylin and eosin, the Van Gieson method, the periodic acid Schiff (PAS) technique, with and without diastase pretreatment, toluidin blue and methyl-green pyronin (M.G.P.).

### RESULTS

### *Regenerating Bone Tissue*

In Table III the composition of regenerating bone tissue studied at various intervals after fracture of the right humerus is indicated.

The morphology of all cells and structures mentioned here is in accordance with the description in textbooks and in the literature.

Osteoprogenitor cells (Young, 1962*a*) are cells on or near the surface of bone or calcified cartilage with inconspicuous cytoplasm and general pale staining nuclei, often oval or fusiform (Fig. 1 and 2).

TABLE III.—*Composition of Regenerating Bone Tissue*

| Days after fracturing | Osteoblasts at periosteal side | Osteoblasts at endosteal side | Osteoid tissue | Trabeculae of bone | Chondroblasts | Hypertrophied chondrocytes | Degenerating chondrocytes | Cartilage inter-cellular substance | Mesenchymal osteoprogenitor cells | Osteoclasts |
|---|---|---|---|---|---|---|---|---|---|---|
| 2 | + | ± | + | — | — | — | — | — | + | — |
| 4 | + | + | + | + | — | — | — | — | + | — |
| 7 | + | + | + | + | + | + | — | + | + | + |
| 10 | + | + | + | + | + | + | + | + | + | + |
| 14 | + | + | + | + | + | + | + | + | + | + |
| 21 | + | + | + | + | — | + | + | + | ± | + |
| 35 | ± | ± | — | + | — | — | — | — | — | + |
| 49 | — | ± | — | + | — | — | — | — | — | + |
| 63 | — | ± | — | ± | — | — | — | — | — | ± |

+ = cells present
— = cells absent
± = questionable

*Histochemical aspects*

*Van Gieson*.—The collagen staining of Van Gieson was positive in the osteoid tissue and in the bone trabeculae; the cartilage intercellular substance stained weaker.

*Toluidine blue*.—The cartilage intercellular substance was strongly metachromatic; the osteoid tissue and bone trabeculae however showed no metachromatic material.

*PAS*.—Mainly in the hypertrophic chondrocytes, glycogen was present. The chondroblasts and degenerating chondrocytes showed much less glycogen.

The various intercellular substances contained PAS positive diastase-resistant material.

*MGP* (Methyl-Green-Pyronin).—The osteoblasts and chondroblasts clearly showed pyroninophilic substance in their cytoplasm. Those osteoprogenitor cells differentiating into chondroblasts and osteoblasts also had this substance, which suggests RNA synthesis by these proliferating and differentiating cells.

In this staining osteoclasts were very difficult to distinguish; therefore it is not clear whether they have pyroninophilic substance.

The osteocytes and degenerating chondrocytes showed no pyroninophilic substance and the hypertrophied chondrocytes only a small quantity of it.

The data concerning the enzyme histochemical investigations of the various cell types are summarized in Table IV.

Although the number of cell types varied during the phases of the fracture healing, they nevertheless showed the same enzyme pattern.

*Alkaline phosphatase*.—The osteoblasts showed a strong enzyme activity; the activity in the osteocytes was low.

The chondroblasts and degenerating chondrocytes varied between a low and moderate activity and the hypertrophied chondrocytes had a moderate activity.

Generally the osteoprogenitor cells showed no enzyme activity.

*Acid phosphatase*.—There was a strong activity in the osteoclasts. The osteoprogenitor cells, the osteoblasts and the cartilage cells showed weak activity. Between the osteoprogenitor cells there were some cells with a moderate activity, just like some fusiform cells between the bone trabeculae; these cells also showed the same activity for $\beta$-hydroxybutyric acid dehydrogenase and succinic acid dehydrogenase and possibly are precursors of osteoclasts.

*ATP-ase*.—The osteoblasts showed variations in enzyme activity from moderate to none. The hypertrophied and degenerating chondrocytes varied between low and a trace of activity.

The osteoprogenitor cells had a weak activity whereas it was marked in the endothelial cells. The osteoclasts were very difficult to distinguish, therefore it is questionable whether they have ATP-ase activity. However, recently Severson, Tonna and Pavelec (1967) showed distinct ATP-ase activity in the osteoclasts.

*5-Nucleotidase*.—In osteoblasts the enzyme activity varied between low and none and in the chondroblasts between moderate and none. The hypertrophied and degenerating chondrocytes showed variations between low and moderate activity and the osteoprogenitor cells between a trace or none. Also in this staining the osteoclasts were indistinguishable, so they probably have the same activity as the osteoblasts.

*NADH-tetrazolium reductase*.—The osteoblasts and osteoprogenitor cells both

TABLE. IV.—*Enzyme Histochemical Pattern of the Various Cell Types in Regenerating Bone Tissue.*

| | Osteoblasts at periosteal and endosteal side | Osteocytes | Chondroblasts | Hypertrophied chondrocytes | Degenerating chondrocytes | Mesenchymal osteoprogenitor cells | Osteoclasts |
|---|---|---|---|---|---|---|---|
| Alkaline phosphatase . . | +++ | −/± | +/++ | ++ | +/++ | −/± | − |
| Acid phosphatase . . . | ±/+ | ± | + | + | + | ±/++ | +++ |
| ATP-ase . . . . | −/++ | − | − | ±/+ | ±/+ | −/± | # |
| 5-Nucleotidase . . . | −/+ | − | −/++ | +/++ | +/++ | −/± | # |
| NADH-tetrazolium reductase | +/++ | ± | +++ | +++ | ++/+++ | ±/++ | ++/+++ |
| NADPH-tetrazolium reductase | +/++ | ± | +++ | +++ | ++/+++ | ±/++ | ++/+++ |
| β-Hydroxybutyric acid dehydrogenase . . . | − | − | −/± | −/± | −/± | −/++ | +++ |
| Succinic acid dehydrog. . | +/++ | ± | ++ | +++ | + | ±/++ | +++ |
| Isocitric acid dehydrog. . | ±/+ | ± | +/++ | +/++ | +/++ | −/± | # |
| Lactic acid dehydrog. . . | ±/++ | ± | ++ | +++ | +/++ | ±/++ | ++/+++ |

−, ±, +, ++, +++, ++++ = no, slight, low, moderate, strong and very strong activity, respectively; / = varying activity. # = see text.

showed moderate but also less activity. The cartilage cells and osteoclasts in general were remarkable for their strong reaction.

*NADPH-tetrazolium reductase.*—This reaction gave the same results as the NADH-tetrazoliumreductase.

*β-Hydroxybutyric acid dehydrogenase.*—Except the osteoclasts, which showed a strong activity, all the cells were practically negative.

Some fusiform cells, however, did show activity; probably these cells are precursors of osteoclasts.

*Succinic acid dehydrogenase.*—The osteoblasts and osteoprogenitor cells again showed practically the same moderate to low activity. The cartilage cells showed variations between strong, moderate and low activity. The osteoclasts had a strong activity and some osteoprogenitor cells were moderate in activity.

*Isocitric acid dehydrogenase.*—A weak activity was found in the osteoblasts, osteocytes and osteoprogenitor cells. Generally the cartilage cells showed more activity. The osteoclasts were indistinguishable from the other cells and it is not clear whether they have activity (Fig. 3).

*Lactic acid dehydrogenase.*—The proliferating osteoblasts and the osteoprogenitor cells varied in enzyme activity between moderate and a trace. The activity of the chondroblasts was moderate, of the hypertrophied chondrocytes strong and of the degenerating chondrocytes varying between low and moderate. The osteoclasts showed a strong to moderate activity (Fig. 4).

### *Induction of Bone Tumours with Radioactive Calcium* ($^{45}$Ca)

Sarcomas of the skeleton—all osteosarcomas—developed in 58 of the 110 mice in a period of 11–19 months. In 6 mice, more than 1 malignant mesenchymal tumour was found: 3 mice had 2 primary osteosarcomas, 2 had next to an osteosarcoma a fibrosarcoma of the soft tissues. The sixth mouse had 3 primary osteosarcomas. There were also some other tumours: benign lung adenomas were found, irrespective of the development of bone tumours, in 24 mice, chiefly among older ones. In $A_2G$ mice the development of lung adenomas is not related to $^{45}$Ca injections (Hadders and Woldring, unpublished data).

One animal had a carcinoma of the breast, a second a fibrosarcoma of the soft tissues and a third a pathological fracture, due to a haemangioma cavernosum in the tibia. These three mice had no osteosarcomas.

In Table V the location of the osteosarcomas and their metastases is summarized.

TABLE V.—*Localization of 63 Primary Osteosarcomas 7 of which Metastasized to the Liver*

| Localization | Number of tumours in 58 mice | Number of mice with hepatic metastases |
|---|---|---|
| Thoracic vertebrae | 13 | — |
| Lumbar vertebrae | 11 | — |
| Sacral vertebrae | 6 | — |
| Pelvis | 10 | 2 |
| Femur | 10 | 5 |
| Tibia | 4 | — |
| Humerus | 2 | — |
| Cranium | 2 | — |
| Ribs | 5 | — |

In general the bone tumours showed infiltration into the surrounding muscles and the tumours of the spine into the spinal cord. The size of the tumours varied from 1 mm. to 3 cm.

Histological investigations of the bone tumours showed osteosarcomas (Fig. 5 and 6). In addition to polymorph plump round cells, resembling osteoblasts, fusiform cells, which showed various mitoses were seen. Sometimes the tumour cells were anaplastic. A few small areas of swollen tumour cells were seen, resembling in a way cartilage. However, the scanty intercellular substance lacked the characteristics of true cartilage.

The quantity of intercellular substance varied from tumour to tumour. Practically no osteoid tissue was found in undifferentiated osteosarcomas in which, besides fusiform and anaplastic tumour cells, giant cells with numerous nuclei were present.

On the other hand tumours with a considerable quantity of osteoid and bone trabeculae were found. These tumours were composed of many polymorph osteoblastic cells, but also osteoclast-like and fusiform cells were present; however, the formation of osteoid in the periphery was scanty while no bone trabeculae were seen. Here, chiefly fusiform and oval tumour cells with various mitoses were found. Due to the bone trabeculae these tumours were clearly visible on the X-ray photographs.

Only in tumours of this kind were hepatic metastases found (Fig. 7 and 8). The hepatic metastases varied in size between 1 mm. and 1 cm; in 1 case practically all liver tissue was replaced by tumour. Histological examination of the metas-

---

### EXPLANATION OF PLATES.

FIG. 1.—Area of a fracture callus. Osteoblasts and osteoclasts between numerous bone trabeculae; osteoprogenitor cells can also be distinguished. H. and E. × 140.

FIG. 2.—Fracture area. Osteoprogenitor cells with oval or fusiform nuclei in the fracture site. H. and E. × 350.

FIG. 3.—Isocitric acid dehydrogenase. The osteoblasts and osteocytes show a varying activity. The osteoclasts are not clearly distinguishable (compare with Fig. 4). × 350.

FIG. 4.—Lactic acid dehydrogenase. Remarkably strong activity in the osteoclasts; the activity in the osteoblasts and osteocytes varies between moderate and a trace. ×350.

FIG. 5.—Osteosarcoma. A considerable quantity of bone trabeculae and numerous polymorph osteoblast-like tumour cells can be distinguished. In addition, polymorph fusiform and oval tumour cells are visible. H. and E. × 140.

FIG. 6.—Osteosarcoma. On the left side sparse, on the right side many, new-formed bone trabeculae. H. and E. × 140.

Fig. 7 and 8.—X-ray photographs of an osteosarcoma with hepatic metastases.

FIG. 9.—Isocitric acid dehydrogenase. Osteosarcoma. Strong activity is visible in the osteoclast-like giant tumour cells. The other tumour cells show less activity. ×350.

FIG. 10.—Alkaline phosphatase. Hepatic metastases of an osteosarcoma. The metastases show a strong activity, even single tumour cells can be distinguished. The endothelium of the vessels also has some activity. × 56.

FIG. 11.—Fibrosarcoma. Polymorph fusiform cells with nucleoli in the hyperchromatic nuclei and mitoses are visible. H. and E. × 350.

FIG. 12.—Osteosarcoma, with scarcely any osteoid, infiltrating muscle. The tumour cells are mainly fusiform or anaplastic. A number of muscle fibres are visible. H. and E. ×140.

Fig. 13.—Fibrosarcoma infiltrating muscle. Here also chiefly fusiform cells are seen. H. and E. × 140.

FIG. 14.—Alkaline phosphatase. Osteosarcoma, the same tumour as in Fig. 12, with strong reaction in the tumour cells. × 140.

FIG. 15.—Alkaline phosphatase. Fibrosarcoma, the same tumour as in Fig. 13. Here there is no activity in the tumour cells. Only the endothelium of the vessels shows some activity. × 140.

1

2

Timmer, Hadders, Hardonk and Koudstaal.

3

4

Timmer, Hadders, Hardonk and Koudstaal.

5

6

Timmer, Hadders, Hardonk and Koudstaal

7

8

9

Timmer, Hadders, Hardonk and Koudstaal.

10

11

12

Timmer, Hadders, Hardonk and Koudstaal.

13

14

15

Timmer, Hadders, Hardonk and Koudstaal.

tases showed bone trabeculae, especially in the centre of the metastasis, while at the edge undifferentiated cells predominated with only a small amount of osteoid tissue.

*Van Gieson.*—This staining showed distinct collagen fibres; even in the most undifferentiated osteosarcomas collagen fibres were found.

*Toluidine blue.*—Metachromatic material was found scantily in osteoid tissue, bone trabeculae and in cartilage-like areas.

*PAS.*—The PAS reaction showed positive material in osteoid tissue and bone trabeculae; glycogen could not be demonstrated.

*Pyroninophilic substance (MGP).*—Both the osteoblast-like tumour cells and the giant cells showed pyroninophilic substance; the undifferentiated tumour cells contained a varying amount of pyroninophilic substance.

In Table VI the enzyme histochemical investigations of the osteosarcomas are summarized.

TABLE VI.—*Enzyme Pattern of the Tumour Cells in Osteosarcomas induced with $^{45}Ca$*

| | Osteoblastic tumour cells | Tumour cells enclosed by intercellular substance | Undifferentiated tumour cells | Giant tumour cells |
|---|---|---|---|---|
| Alkaline phosphatase . | +++ | + | −/+++ | − |
| Acid phosphatase . | ±/++ | ± | ±/++ | +++/++++ |
| ATP-ase . . . | −/++ | − | +/++ | − |
| 5-Nucleotidase . . | −/+ | − | −/+ | ±/++ |
| NADH-tetrazolium reduct. | +/++ | ± | +/++ | +++ |
| NADPH-tetrazolium reduct. | +/++ | ± | +/++ | +++ |
| β-Hydroxybutyric acid dehydrogenase . . | − | − | − | ++ |
| Succinic acid dehydrogenase | +/++ | ± | +/++ | +++ |
| Isocitric acid dehydrogenase | ±/+ | ± | ±/+ | ++/+++ |
| Lactic acid dehydrogenase | + | ± | ++ | +++ |

−, ±, +, ++, +++, ++++ = no, slight, low, moderate, strong, very strong activity, respectively; / = varying activity.

*Alkaline phosphatase.*—The osteoblastic tumour cells were strongly positive, while the giant cells did not show a trace of activity. The undifferentiated tumour cells showed varying activity: the cells in the periphery of an osteoid and bone trabeculae forming osteosarcoma, and the cells in an undifferentiated osteosarcoma clearly showed a strong activity. The undifferentiated cells between the bone trabeculae scarcely showed any activity.

*Acid phosphatase.*—A strong to very strong activity was found in the giant tumour cells.

The osteoblastic and undifferentiated tumour cells showed a moderate to low activity.

*ATP-ase.*—The osteoblastic tumour cells were moderate positive to negative. The giant cells were negative and the undifferentiated tumour cells showed a moderate to a low activity. The endothelium of the vessels was strongly positive.

*5-Nucleotidase.*—In the osteoblastic and undifferentiated tumour cells a low activity was found, which was somewhat higher in the giant tumour cells.

*NADH-tetrazolium reductase.*—The osteoblastic and undifferentiated tumour cells showed a low to moderate activity, the giant tumour cells were strongly positive.

*NADPH-tetrazolium reductase.*—This enzyme reaction gave the same results in the tumour cells as the NADH-tetrazolium reductase reaction.

*β-Hydroxybutyric acid dehydrogenase.*—Apart from a remarkable activity in the giant tumour cells, no activity was found in the other tumour cells.

*Succinic acid dehydrogenase.*—A low to moderate activity was found in the osteoblastic and undifferentiated tumour cells; the giant tumour cells however were strongly positive.

*Isocitric acid dehydrogenase.*—In contrast to normal osteoclasts, the giant tumour cells showed a moderate to strong activity (Fig. 9). A weak reaction was visible in the osteoblastic and undifferentiated tumour cells.

*Lactic acid dehydrogenase.*—A low activity was found in the osteoblastic tumour cells, while in the undifferentiated tumour cells it was moderate. The giant tumour cells showed, as in the other dehydrogenase reactions, a remarkable activity.

*Metastases*

Generally the enzyme histochemical pattern of the cells in the tumour metastases showed no important variation from that of the corresponding cells of the primary tumour.

The alkaline phosphatase showed strong activity especially in the periphery of the metastasis, and thus in undifferentiated tumour cells. Sometimes single tumour cells were seen in the lumen of the vessels, near the tumour metastasis (Fig. 10).

### *Induction of Tumours with 20-MC*

It was impossible to induce tumours originating from the skeleton by application of 20-MC in the callus of a fractured bone.

The induced tumours in groups X, XI and XII (Table II) were fibrosarcomas; nowhere was formation of bone, osteoid or cartilage found. It was supposed that the tumours originated from the lesions in the soft tissues.

To prove this supposition lesions were made only in the muscles and not in the bone of the upper right leg (groups XVI, XVII and XVIII) after which 20-MC was injected. Here also sarcomas developed which histologically could not be distinguished from the tumours of groups X, XI and XII.

In 17 of 25 mice of group X fibrosarcomas developed in the course of 79 days in the soft tissues only. No tumour was in contact with the area of the fracture and always there was some normal soft tissue between tumour and bone.

On the left side no tumours were seen at all. The tumours varied in size between 2 mm. and 4 cm., infiltrating the surrounding muscles and fat tissue. The tumours were composed of polymorph, fusiform mesenchymal cells, with hyperchromatic nuclei and clear nucleoli; various mitoses were seen (Fig. 11). The intercellular matrix consisted of fibres of varying thickness to broad collagen bundles. The results are summarized in Table VII.

In groups XI and XII tumours arose not only along the right fractured humerus but also along the left intact humerus probably as a result of destruction of muscle tissue during the injection.

In the control groups injected with oleum arachii no tumour developed.

It was remarkable that the greatest number of tumours was seen in groups XVI, XVII and XVIII, many of which were bilateral.

TABLE VII.—*Induction of Tumours with 20-MC in Fractured and Intact Legs and in Lacerated Muscles*

| | Groups of 25 male mice | Tumour of right humerus | Tumour of left humerus | Tumour of right and left humeri | No tumour |
|---|---|---|---|---|---|
| (x) | X | 17 | — | — | 8 |
| | XI | 8 | — | 3 | 14 |
| | XII | 8 | 3 | 5 | 9 |
| | XIII | — | — | — | 25 |
| | XIV | — | — | — | 25 |
| | XV | — | — | — | 25 |
| (x) | XVI | 3 | 6 | 13 | 3 |
| (x) | XVII | 7 | 4 | 12 | 2 |
| (x) | XVIII | 5 | 3 | 15 | 2 |

(x) One carcinoma planocellulare of the skin was present in this group.

Fibrosarcomas of the soft tissues were induced after injecting 20-MC, first into a fracture of the right humerus (X, XI, XII, secondly of the left humerus (X, XI, XII, XVI, XVII, XVIII) and thirdly into lacerated muscles of the right upper leg (XVI, XVII, XVIII).

Control groups XIII, XIV and XV had 2 injections of oleum arachii without 20-MC into a fracture of the right humerus and along the left intact humerus.

### *Histochemical results*

*Van Gieson.*—The intercellular substance showed collagen fibres.

*Toluidine blue.*—There was scarcely any metachromatic material in the intercellular substance.

*PAS.*—The intercellular substance was positive; glycogen was not demonstrable in the tumour cells.

*MGP.*—The tumour cells showed a lot of pyroninophilic substance in their cytoplasm.

The enzyme histochemical results of the fibrosarcomas are summarized in Table VIII.

TABLE VIII.—*Enzyme Histochemical Pattern of Fibrosarcomas Induced with 20-MC in Fractured and Intact Legs and in Lacerated Muscles*

| Oval or fusiform tumour cells. | |
|---|---|
| Alkaline phosphatase | — |
| Acid phosphatase | +/++ |
| ATP-ase | +/++ |
| 5-Nucleotidase | −/+ |
| NADH-tetrazolium reductase | +/+++ |
| NADPH-tetrazolium reductase | +/+++ |
| β-Hydroxybutyric dehydrogenase | — |
| Succinic acid dehydrogenase | +/+++ |
| Isocitric acid dehydrogenase | +/++ |
| Lactic acid dehydrogenase | +/+++ |

−, ±, +, ++, +++, ++++ = no, trace, moderate, strong, very strong activity, respectively; / = varying activity.

The various tumours did not show important enzyme histochemical variations.

*Alkaline phosphatase.*—No activity was seen in the tumour cells of the fibrosarcoma, which is in contrast to the cells of the osteosarcoma.

*Acid phosphatase.*—The tumour cells varied between moderate activity and a trace of activity.

*ATP-ase.*—Like the undifferentiated tumour cells of the osteosarcoma the cells showed a moderate to low activity.

Strong activity in the endothelium of the vessels also was present in the fibrosarcoma.

5-*Nucleotidase.*—The tumour cells scarcely showed any activity.

*Dehydrogenase reactions.*—Generally the tumour cells showed a remarkable activity in the various dehydrogenase reactions with the exception of $\beta$-hydroxybutyric acid dehydrogenase, which was absent in the tumour cells.

### DISCUSSION

In normal bone there is a dynamic equilibrium between formation and resorption of bone. In healing fractures the former temporarily exceeds the latter. After some time, in mice about after 60 days, the balance is restored. In the bone tumour on the other hand there is an irreversible disturbance of this equilibrium. The cells responsible for the process of bone formation are the osteoblasts. Nowadays it is generally accepted that the osteoclasts play an important active role in the resorption of bone. Both types of cells are derived from undifferentiated mesenchymal cells.

The term differentiation is used by us for the process by which the cell is committed to a final form and function.

Young (1962*b*) investigated the proliferation and differentiation of bone cells and concluded that the various types of bone cells represent different functional states of the same cell and that cell division is restricted to undifferentiated " osteoprogenitor cells ". These are cells on or near the surface of bone or calcified cartilage with inconspicuous cytoplasm and general pale staining nuclei, often oval or fusiform.

As the term mesenchymal cell implies a vast range of possible differentiations the more restrictive term osteoprogenitor cell has been used for cells from which bone and cartilage-forming cells are derived.

In respect of our experiments the most important groups of cells which arise from the primitive mesenchymal cells are given in Table IX.

TABLE IX.—*Differentiation of Mesenchymal Cells into Bone and Connective Tissue Forming Cells.*

In our experiment the differentiation of osteoprogenitor cells was disturbed by $^{45}Ca$, resulting in bone tumours. These tumours consisted of undifferentiated polymorph cells, giant cells and polymorph osteoblastic cells. In the latter we found, just as in the osteoblasts of regenerating bone, a strong activity of alkaline phosphatase. All tumour cells in the undifferentiated osteosarcomas and the undifferentiated tumour cells in the periphery of bone-forming osteosarcomas and hepatic metastases also were strongly positive. In these areas many mitoses were seen, while any osteoid was present.

Many opinions are held about the role of alkaline phosphatase. Robison (1923) and Robison and Rosenheim (1934) stated that alkaline phosphatase is of importance in the calcification mechanism. Fell and Danielli (1943) suggested that this enzyme is important in the mechanism of synthesis of a fibrous protein. Siffert (1951) concluded that phosphatase is related to preosseous matrix formation and that the elaboration of osteoid, which may occur in the absence of calcification, is always associated with phosphatase activity. In 1964 Beneke demonstrated *in vitro* precipitation of normal serum proteins by mucopolysaccharides. Lipp (1967) showed with fluorochrome-labelled homologous blood serum that certain serum components are concentrated and incorporated into the organic bone matrix during its formation. This concentration and incorporation was not found in experimental rickets. The results of these experiments are interpreted in terms of a calcium-carrying serum protein being complexed and precipitated by products of osteoblasts, probably mucopolysaccharides, to form an essential component, which possibly initiates nucleation and the subsequent steps of calcification.

Possibly the alkaline phosphatase in the osteoblasts produces a high concentration of phosphate ions, which may be bound by the calcium in the bone matrix, resulting in calcification.

Thus the intercellular substance, which is formed by the undifferentiated highly alkaline phosphatase active tumour cells, may not be able to precipitate calcium-carrying serum proteins. The phosphate ions produced by the alkaline phosphatase in the tumour cells cannot be bound, and calcification does not occur.

The alkaline phosphatase activity of these cells contrasts with the undifferentiated osteoprogenitor cells in the healing fracture, which showed no alkaline phosphatase activity except when differentiating to osteoblasts and chondroblasts. In regenerating bone tissue of a fracture, the alkaline phosphatase positive cells (osteoblasts) are considered as non-dividing differentiated cells. So in the tumour there are undifferentiated, dividing cells with the same alkaline phosphatase activity as the highly differentiated non-dividing cells in fracture healing.

A combination of alkaline phosphatase and autoradiography by means of tritiated thymidine on the same section of an osteosarcoma could possibly make sure whether the undifferentiated tumour cells can divide and differentiate at the same time.

In the centre of the bone-forming osteosarcomas, the undifferentiated tumour cells between the bone trabeculae scarcely showed any activity of alkaline phosphatase, just as do the osteoprogenitor cells in the healing fracture.

As it was very difficult to estimate the alkaline phosphatase activity of the osteoclasts in regenerating bone tissue, a combined alkaline phosphatase acid phosphatase reaction was carried out which showed generally no alkaline phosphatase activity in the osteoclasts.

Except for $\beta$-hydroxybutyric acid dehydrogenase the osteoblasts, the osteoblastic and undifferentiated tumour cells showed clear dehydrogenase activity; also isocitric acid dehydrogenase activity was present, which might indicate production of glutamate from pyruvate according to Walker (1961).

The normal osteoclasts and giant tumour cells clearly showed strong activity of acid phosphatase. At present it is assumed that this enzyme plays an important role in the process of phagocytosis. Therefore, the presence of acid phosphatase in the osteoclasts and giant tumour cells could indicate the possibility that these cells are phagocytic.

In the osteoclasts the activity of $\beta$-hydroxybutyric acid, succinic acid and lactic acid dehydrogenase was very high; isocitric acid dehydrogenase activity however was difficult to demonstrate.

The same results were reported by Walker (1961) who concluded that the Krebs cycle in the osteoclast is changed. He suggested that citric acid and lactic acid are produced from some of the principle constituents of collagen. These acids are able to dissolve calcium from the bone matrix. On the other hand some authors (Fullmer, 1963; Balogh, Dudley and Cohen, 1961; Balogh and Hajek, 1965; Takada, 1966) could show a strong isocitric acid dehydrogenase activity in the osteoclasts. The experiments of these authors, however, were carried out with rats and also with young mice. No decalcification of the latter was necessary. We could not confirm their findings in our experiment. As for the giant tumour cells we found remarkably strong activity of isocitric acid dehydrogenase, so it is possible that the presence of this enzyme decreases the citrate concentration which is necessary for decalcification of bone tissue. The giant tumour cells, although rich in acid phosphatase, cannot phagocytose bone tissue which is not decalcified. It can be concluded that normal regenerating bone differs histologically as well as enzyme-histochemically from the bone formation in an osteosarcoma.

We failed to induce bone tumours by injecting a solution of 20-MC in oleum arachii. On the contrary Yamada (1965) succeeded in inducing bone tumours by the implantation of crystals of 20-MC in a fracture callus. In our experiments the fibrosarcomas arising from the soft tissues were probably the result of the influence of 20-MC on the granulation tissue, which developed in the soft tissues after the fracture of the bone and/or after the injection. This could be proved in the experiments XVI, XVII and XVIII, where only the soft tissues were destroyed. It was striking that most tumours arose in groups XVI, XVII and XVIII, and not only in the lacerated muscles but also on the left side. Possibly the damage caused by the injection alone is sufficient for the application of 20-MC to induce fibrosarcomas. A clear histological difference between the highly undifferentiated osteosarcoma and the fibrosarcoma was sometimes very difficult to demonstrate (Fig. 12 and 13). Enzyme histochemical methods, however, solved these problems, as the most striking point of difference between these tumours in mice was the alkaline phosphatase reaction (Fig. 14 and 15).

### SUMMARY

Fracture healing was used for studying the regeneration of bone; histological and enzyme histochemical investigations were carried out.

The formation of normal bone was compared with the pathological bone of osteosarcomas caused by injecting female $A_2G$ mice with $^{45}Ca$.

Enzyme histochemical, as well as histological, differences were found, particularly in regard to the activity of isocitric acid dehydrogenase and alkaline phosphatase. Finally, the osteosarcomas were compared with fibrosarcomas arising from soft tissue after injecting 20-methylcholanthrene into fracture callus and into lacerated muscles.

Here also histological and enzyme histochemical differences were present. Of these the most important was the alkaline phosphatase activity, which was strong in the osteosarcomas and negative in the tumour cells of the fibrosarcomas.

The authors wish to acknowledge the valuable technical assistance of Mr. P. K. Houwing and Mr. L. Smid in performing the staining reactions and Miss B. Wassing in preparing the manuscript.

The photography was done in the photographic division of the Department of Pathology (J. J. Wachters).

We are greatly indebted to Dr. M. G. Woldring, who was helpful in administering $^{45}$Ca.

### REFERENCES

ANDERSON, W. A. D., ZANDER, G. E. AND KUZMA, J. F.—(1956) *Archs Path.*, **62**, 262.
BALOGH, K., DUDLEY, H. R. AND COHEN, R. B.—(1961) *Lab. Invest.*, **10**, 839.
BALOGH, K. AND HAJEK, J. V.—(1965) *Am. J. Anat.*, **116**, 429.
BARKA, T. AND ANDERSON, P. J.—(1962) *J. Histochem. Cytochem.*, **10**, 741.
BARNES, J. M., DENZ, F. A. AND SISSONS, H. A.—(1950) *Br. J. Cancer*, **4**, 212.
BARNES, L. L., SPERLING, G., MCCAY, C. M. AND BROWN, C. E.—(1958) *Archs Path.*, **66**, 529.
BASERGA, R., LISCO, H. AND CATER, D. B.—(1961) *Am. J. Path.*, **39**, 455.
BENEKE, G.—(1964) *Verh. dt. Ges. Path.*, **48**, 306.
BRUNSCHWIG, A.—(1938) *Am. J. Cancer*, **34**, 540.
BURSTONE, M. S.—(1961) *J. Histochem. Cytochem.*, **3**, 146.
CLOUDMAN, A. M., VINING, D., BARKULIS, S. AND NICKSON, J. J.—(1949) *Am. J. Path.*, **25**, 810.
DUNLAP, C. E., AUB, J. C., EVANS, R. D. AND HARRIS, R. S.—(1944) *Am. J. Path.*, **20**, 1.
DUSTRA, F. R. AND LARGENT, E. J.—(1950) *Am. J. Path.*, **26**, 197.
FELL, H. B. AND DANIELLI, J. F.—(1943) *Br. J. exp. Path.*, **24**, 196.
FINKEL, M. P., BERGSTRAND, P. J. AND BISKIS, B. O.—(1961) *Radiology*, **77**, 269.
FINKEL, M. P. AND BISKIS, B. O.—(1962) *Hlth Phys.*, **8**, 565.
FINKEL, M. P., BISKIS, B. O. AND JINKINS, P. B.—(1966) *Science, N.Y.*, **151**, 698.
FULLMER, H. M.—(1966) *Annls Histochim.*, **11**, 369.
GARDNER, L. U. AND HESLINGTON, H. F.—(1946) *Fedn Proc. Fedn Am. Socs exp. Biol.*, **5**, 221.
GRAFFI, A.—(1960) *Arch. klin. Chir.*, **295**, 40.
GROSS, L.—(1953) *Proc. Soc. exp. Biol. Med.*, **83**, 414.
HARDONK, M. J..—(1965) *Histochemie*, **4**, 563.
JANES, J. M.—(1956) *J. Bone Jt. Surg.*, **38A**, 809.
KIRSTEN, W. H., ANDERSON, D. G., PLATZ, C. E. AND CROWELL, E. B.—(1962) *Cancer Res.*, **22**, 484.
KUZMA, J. F. and ZANDER, G.—(1957) *Archs Path.*, **63**, 198.
LIPP, W.—(1967) *Histochemie*, **9**, 339.
MARKOWA, J. AND MARCK, A.—(1967) *Nature, Lond.*, **213**, 831.
MORI, M., ITO, M. AND FUKUI, S.—(1965) *Histochemie*, **5**, 185.
NACHLAS, M. M., TSON, K. C., DE SONZA, E., CHERY, C. S. AND SELIGMAN, A. M.—(1957) *J. Histochem. Cytochem.*, **5**, 420.

NACHLAS, M. M., WALKER, D. G. AND SELIGMAN, A. M.—(1958) *J. biophys. biochem. Cytol.*, **4**, 29, 467.
OWEN, M., SISSONS, H. A. AND VAUGHAN, J.—(1957) *Br. J. Cancer*, **11**, 229.
ROBISON, R.—(1923) *Biochem. J.*, **17**, 286.
ROBISON, R. AND ROSENHEIM, A. H.—(1934) *Biochem. J.*, **28**, 684.
SEVERSON, A. R., TONNA, E. A. AND PAVELEC, M.—(1967) *J. Histochem. Cytochem.*, **15**, 550.
SIFFERT, R. S.—(1951) *J. exp. Med.*, **93**, 415.
SKORYNA, S. C. AND KAHN, D. S.—(1959) *Cancer, N.Y.*, **12**, 306.
STANTON, M. F.,—(1967) *Cancer Res.* **27**, 1000.
TAKADA, K.—(1966) *Acta histochem.*, **23**, 53.
TAPP, E.—(1966) *Br. J. Cancer*, **20**, 778.
WACHSTEIN, M. AND MEISEL, E.—(1957) *Am. J. clin. Path.*, **27**, 13.
WALKER, D. G.—(1961) *Johns Hopkins Hosp. Bull.*, **108**, 80.
WARREN, S. AND CHUTE, R. W.—(1963) *Lab. Invest.*, **12**, 1041.
YAMADA, M.—(1965) *Excerpta med., Cancer*, **13**, 989.
YOUNG, R. W.—(1962*a*) *Expl cell. Res.*, **26**, 562.—(1962*b*) *J. cell. Biol.*, **14**, 357.